# Genome-Wide Association Study Reveals a New Quantitative Trait Locus in Rice Related to Resistance to Brown Planthopper *Nilaparvata lugens* (Stål)

**DOI:** 10.3390/insects12090836

**Published:** 2021-09-17

**Authors:** Longqing Shi, Meng Dong, Ling Lian, Junian Zhang, Yongsheng Zhu, Weilong Kong, Liangmiao Qiu, Dawei Liu, Zhenxing Xie, Zhixiong Zhan, Zhaowei Jiang

**Affiliations:** 1 Rice Research Institute, Fujian Academy of Agricultural Sciences, Cangshan, Fuzhou 350018, China; shilongqing@faas.cn (L.S.); dongmeng@faas.cn (M.D.); lianling@faas.cn (L.L.); zhangjunian@faas.cn (J.Z.); zhuyongsheng@faas.cn (Y.Z.); liudawei@faas.cn (D.L.); xiezhenxing@faas.cn (Z.X.); 2 Shenzhen Branch, Guangdong Laboratory of Lingnan Modern Agriculture, Genome Analysis Laboratory of the Ministry of Agriculture and Rural Affairs, Agricultural Genomics Institute at Shenzhen, Chinese Academy of Agricultural Sciences, Shenzhen 518120, China; kongweilong@caas.cn; 3 Institute of Plant Protection, Fujian Academy of Agricultural Sciences, Fuzhou 350013, China; bjndqlm@163.com

**Keywords:** durable resistance, GWAS, Host-plant resistance, resistance genes, rice pest

## Abstract

**Simple Summary:**

The brown planthopper *Nilaparvata lugens* (Stål) (BPH) is one of the main rice pests in Asian areas. The development of rice varieties harboring resistance genes is the most economical and effective method of managing BPH. In this study, 123 rice germplasms were identified for resistance and durable resistance by using the rice planthopper resistance identification system. Forty-two of the 123 rice varieties were classified as resistant to brown planthopper, and among them, twelve rice varieties had a long, durable resistance period. One potential durable resistance to brown planthopper locus on chromosome 2 was found by a genome-wide association study (GWAS). There are 13 candidate genes at this locus, and several of them are related to disease and pest resistance. Our study found a potential durable resistance locus to BPH, which has guiding significance for subsequent resistance breeding.

**Abstract:**

The brown planthopper (BPH) is one of the main pests endangering rice yields. The development of rice varieties harboring resistance genes is the most economical and effective method of managing BPH. To identify new BPH resistance-related genes, a total of 123 rice varieties were assessed for resistance and durable resistance. Three varieties were immune, and nine were highly resistant to BPH. After whole-genome resequencing of all 123 varieties, 1,897,845 single nucleotide polymorphisms (SNPs) were identified. Linkage disequilibrium (LD) decay analysis showed that the average LD of the SNPs at 20 kb was 0.30 (r^2^) and attenuated to half value (~0.30) at a distance of about 233 kb. A genome-wide association study (GWAS) of durable resistance to BPH was conducted using the Fast-MLM model. One quantitative trait locus, identified on chromosome 2, included 13 candidate genes. Two candidate genes contained a leucine-rich repeat and CC-NBS-LRR or NB-ARC domains, which might confer resistance to pests or diseases. Interestingly, *LOC_Os02g27540* was highly expressed and was induced by BPH; GWAS identified potential rice genes coding for durable resistance to BPH. This study helps to elucidate the mechanism of durable resistance to BPH in rice and provides essential genetic information for breeding and functional verification of resistant varieties.

## 1. Introduction

Rice (*Oryza sativa*) is the leading food for nearly half the world’s population and about 90% of it is produced in Asian countries [1]. Rice yield is affected by many abiotic or biotic stress, but pests are one of the main factors. The brown planthopper *Nilaparvata lugens* (Stål) (BPH) is a monophagous phytophagous insect that causes severe damage to the rice industry in China and many areas of Asia, with millions of tons lost each year [2,3].

There are three advantages of BPHs that help them become the primary pest in rice fields. Firstly, the adult BPH has prominent wing dimorphism. The long-winged adults (macropterous form) can migrate long distances to escape harsh conditions and seek suitable environments for survival and reproduction. Short-winged adults (brachypterous form) cannot fly; compared with the macropterous form, the brachypterous female can reproduce quickly and expand the population [4,5,6]. Secondly, the BPH has a short generation cycle and fast metabolism, which help it to develop resistance to chemical pesticides. To date, the BPH has developed resistance to 31 chemical pesticides, including imidacloprid, dinotefuran, and clothianidin [7]. Thirdly, the BPH inhabits the base of the rice stem near the ground [8]. After the rice tillering stage, the leaves are densely distributed on the upper plant, which blocks pesticides applied through standard spraying methods and reduces the pesticide exposure of the BPH.

Host-plant resistance (HPR) results from animal and plant co-evolution and plays a vital role in integrated pest management [9,10,11]. Resistant host plants can prevent pest damage through physical barriers (waxy layers, epidermal hair, etc.) and through their own, or induced, chemicals that reduce the growth, development, survival, or reproductive fitness of pests [12,13,14]. A practical method to control BPH is to breed and plant rice varieties with improved resistance via chemical, physical, biological, and genetic strategies [15,16]. Both 1–5 instar nymphs and adults of the BPH pierce the phloem of the rice stems and suck the plant sap, which is the primary way the BPH harms the rice plant. Saliva is secreted, forming a sheath-like structure (saliva sheath) conducive to the scalpella withdrawing the sap. The saliva sheath coagulates and remains in the phloem after feeding, blocking nutrient transport [8,17]. BPH-resistant rice varieties have constitutive resistance and induced resistance, preventing the pest from causing further damage after insertion of the mouthparts and saliva [12]. BPH resistance genes are essential in regulating resistance in rice. For example, Bph29 activates the salicylic acid signaling pathway by encoding the B3 DNA-binding protein, inhibiting the jasmonic acid/ethylene-dependent (JA/Et) pathway, and encouraging callose to accumulate in the phloem sieve, which discourages feeding by the BPH [18]. Bph14 and Bph26 are typical CC-NBS-LRR (coiled-coil nucleotide-binding site leucine-rich repeats) resistance genes (R genes) that encode proteins that inhibit the BPH [19,20]. To date, 38 BPH resistance genes have been identified in rice. They are mainly distributed on chromosomes 3, 4, 6, and 12 in the form of gene clusters [21]. Many BPH resistance genes remain to be identified, and the molecular mechanisms of some cloned genes need further studies.

In this study, the BPH resistance and durable resistance periods of the 123 rice varieties at the seedling stage were identified by seedbox screening. Their quantitative trait loci (QTLs) were identified by genome-wide association study (GWAS). The candidate genes were then analyzed based on their gene annotations and were verified by quantitative real-time PCR (qRT-PCR). This study will help discover different resistance genes and the breeding of BPH-resistant rice varieties.

## 2. Materials and Methods

### 2.1. Breeding of BPH

Brown planthoppers were provided by the Rice Research Institute, Fujian Academy of Agricultural Sciences, Fuzhou, China, Fujian Academy of Agricultural Sciences (Fuzhou, China). The strain had been collected from an experimental rice field in 2016 and was reproduced with TN1 rice seedlings for more than 40 generations. The conditions for indoor breeding were 26 ± 2 °C, 65 ± 5% relative humidity, and 14 hours with light(L): 10 h with darkness (D) photoperiod (artificial lighting).

### 2.2. Rice Materials

A total of 123 rice varieties was used in this study. All were hybrid rice parents, most of them were used in rice breeding. Seeds were provided by the Rice Research Institute, Fujian Academy of Agricultural Sciences, Fuzhou, China. To guarantee the high capacity of seed germination, the seeds of 123 rice varieties were planted at the farm of Rice Research Institute of Fujian Academy of Agricultural Sciences in Fuzhou (119°21′57.6″ E, 26°0′46.8″ N). The new harvest seeds were used for the following tests.

### 2.3. Identification of Resistance to Brown Planthopper in Rice Seedlings

The resistance of rice seedlings to BPH was identified by a modified seedbox screening test [22] as follows: Seedling trays (50 × 35 × 8 cm) were covered with soil taken from a paddy field. Soil was evenly divided into 11 rows with one rice variety per row, 12 seedlings per variety, and three biological replicates per variety. Each tray contained ten varieties and the susceptible control TN1 in a random block design. Two plants of each variety were randomly selected at the one-tip-two-leaf stage and stored (without roots) at −80 °C for subsequent sequencing. The remaining ten seedlings of each variety were artificially inoculated with 8–10 nymphs of 1–2 instar BPHs per seedling. Seedling mortality was recorded every day. When the mortality of the susceptible TN1 variety reached 70%, the number of dead seedlings of each variety was recorded daily. The mean resistance grade of three biological replicates of each variety was assessed when all TN1 plants had died. For the varieties with resistance grades of 1 to 5, changes in seedling damage and grading continued to be recorded after the TN1 resistance grade reached 9 (seedling mortality > 70%) to evaluate their durable resistance period (started from the day when 70% of TN1 seedlings were dead, ending 3 days before 70% of the tested variety seedlings were dead) [22].

### 2.4. Whole-Genome Resequencing and Mutation Detection

All 123 rice varieties were sequenced by paired-end sequencing on an Illumina Hiseq2000 sequencing platform (BioMarker, Beijing, China). The clean reads were aligned against the *O. sativa* genome (v7.0) using BWA [23] (https://genome.jgi.doe.gov/portal/pages/dynamicOrganismDownload.jsf?organism=Osativa, 2015). The Genome Analysis Toolkit (GATK v3.8) [24] was used for identifying variants, including SNPs and small InDels. SNPs/Indels were screened, excluding missing values and minimum allele frequencies (MAF) above 0.05. Finally, 3,502,938 high-quality SNPs were screened by GWAS analysis. An SNP density map was generated and analyzed using the CMplot package of R. Principal component analysis (PCA) of this rice population structure was conducted based on the selected high-quality SNP loci using EIGENSOFT.

### 2.5. Genome-Wide Association Analysis of Durable Resistance

The population structure of the 123 varieties was determined based on SNPs using ADMIXTURE software. The number of subgroups (K value) was preset to 1–10 for clustering. The optimal number of clusters was determined according to the minimum of the cross-validation error rate. Based on the developed high-density SNP molecular markers, TASSEL, FaST-LMM, and EMMAX were used for association analysis. The detected SNPs were filtered according to the minor allele frequency (MAF 0.05) and locus integrity (INT 0.8) to obtain a highly consistent SNP locus for downstream analysis. Manhattan plots were generated by the qqman package of R, and Q-Q plots were created to display the results of the association analysis. Linkage disequilibrium analysis of the chromosome peak region was conducted using LDBlockShow.

### 2.6. Transcript Abundance Analysis

**Figure 1:**
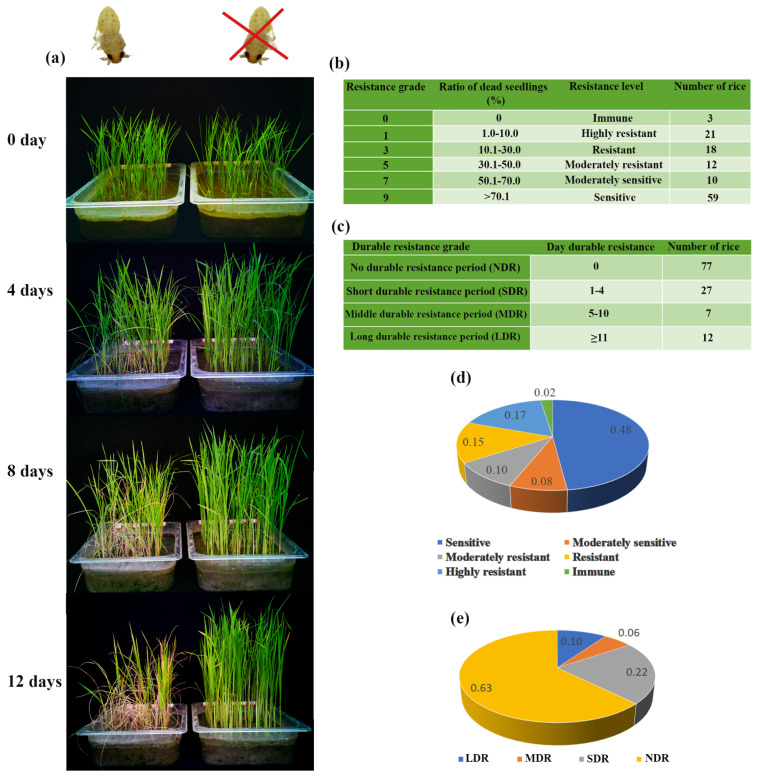
Phenotype identification of rice seedling resistance. (**a**) The phenotypic changes in TN1 rice (sensitive) seedlings inoculated with BPH nymphs after 0, 4, 8, and 12 days (the left tray); the right tray was treated without nymphs as a control. (**b**) Grading of rice seedling resistance to BPH. (**c**) Grading of the rice seedling durable resistance period for BPH. (**d**) Ratios of each resistance grade. (**e**) Ratios of each durable resistance grade.

Seeds of two varieties with a long, durable resistance period (LDR) (IR24 and R26) and two with no durable resistance (NDR) (R01 and R121) were cultured to their one-tip-two-leaf stage (Figure 1a, 0 day). Then, the seedlings were inoculated with 1–2 instar BPH nymphs, 8–10 nymphs per seedling. The stem and leaf of seedlings were sampled 0 days, 4 days, 8 days, and 12 days after the nymph inoculation. All the samples were quick-frozen in liquid nitrogen before being stored in a −80 °C refrigerator. The total RNA was isolated using TRIzol reagent. Then, the cDNA was generated using the RevertAid First Strand cDNA Synthesis Kit (Fermentas, Vilnius, LTU). The qRT-PCR was performed with the Fast Start Universal SYBR Green Master system (Roche, Indianapolis, USA) on a QuantStudio^TM^ 6 Flex System (Applied Biosystems). The relative quantitative method (ΔΔCT) was used to evaluate the quantitative variation in the examined replicates. The statistical calculations and histogram drawing were performed using Microsoft Excel 2019. Statistical analyses were performed using one-way ANOVA or Student’s t tests, and *p*-values < 0.05 were considered to indicate statistical significance. The primers used in qRT-PCR are listed in Table S1.

## 3. Results

### 3.1. Identification of Rice Resistance to BPH

In this study, 123 hybrid rice parents were collected and cultured to their one-tip-two-leaf stage and were then inoculated with 1–2 instar BPH nymphs. Figure 1a displays the phenotype of TN1 (a typical insect-susceptible rice variety). On the 4th day after BPH nymph inoculation, the growth of TN1 rice seedlings (the left tray) was slower and a few of them started to wilt compared with the control (the right tray). This situation became more serious over time, and on the 12th day, almost 90% of seedlings (the left tray) were wilted and dead.

Forty-two of the 123 rice varieties were classified as resistant to BPH (grades 0–3) through phenotypic identification, of which 21 varieties were highly resistant (grade 1, with a ratio of 0.17) and three were immune (grade 0, with a ratio of 0.02) (Figure 1b,d). The durable resistance period was evaluated according to the categories in Figure 1c. Forty-six of the 123 rice varieties exhibited durable resistance to BPH, of which seven had a moderate durable resistance period (MDR, with a ratio of 0.06) and twelve had a long durable resistance period (LDR, with a ratio of 0.10) (Figure 1c,e).

### 3.2. SNP and Population Structure Analysis

**Figure 2:**
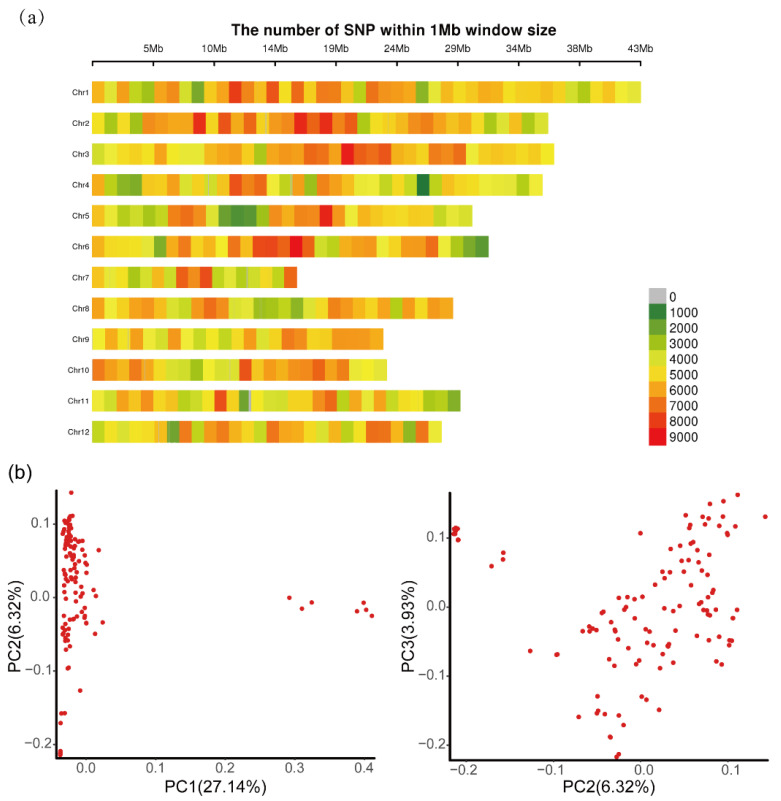
SNP distribution density map on chromosomes and principal component analysis (PCA) of 123 rice varieties based on SNPs. (**a**) The distribution of SNPs on rice chromosomes; the number of SNPs per 0.1 Mb is displayed as a color index (lower right corner); (**b**) Principal component analysis of the genetic variation of the germplasm of 123 rice varieties.

A total of 1237.74 Gb clean data was obtained. All sequence data of the samples were uploaded to the website of the NCBI SRA (Sequence Read Archive) database; the accession number is PRJNA743713. The average mapping rate of all samples on the reference genome was 97.91%. The average coverage depth was 22×, and the genome coverage was 92.58% (Table S2). Genome Analysis Toolkit (gatk) software was used to detect single nucleotide polymorphisms (SNP) in the 123 rice varieties. A total of 1,897,845 SNP sites were detected after subsequent analysis. The distribution of these SNPs in the genome is shown in Table 1 and Figure 2. The average density of SNPs in the genome was 204 bp/SNP. The number of SNPs among the 12 rice chromosomes ranged from 83,242 to 231,924. The smallest average density of SNPs was on chromosome 7 with one SNP per 371 bp, while chromosome 3 exhibited a maximum marker density with one SNP per 180 bp. The number of SNPs among the 12 rice chromosomes ranged from 83,242 to 231,924. The smallest average density of SNPs was on chromosome 7 (371 bp/SNP) and the largest was on chromosome 3 (180 bp/SNP).

PCA was performed based on the high-quality SNPs; all the varieties had no obvious subgroups and most were clustered together, except for a few *japonica* varieties separated from the *indica* varieties (on the first principal component there was a 27.14% contribution to the difference); on the second and third principal components, they were not clustered into clearly defined groups (Figure 2b).

### 3.3. Linkage Disequilibrium Analysis

**Figure 3:**
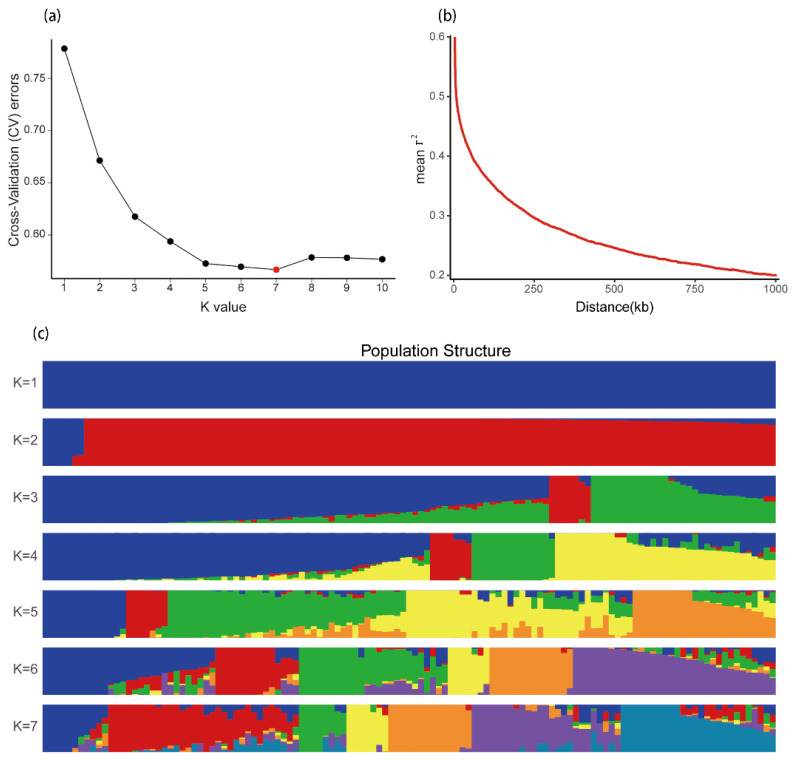
Cross-validation, population linkage disequilibrium attenuation, and ADMIXTURE analysis of genetic relationships. (**a**) Cross-validation (CV) scores at different K values. The lowest value (K = 7) was selected as optimal for ADMIXTURE analysis. (**b**) Average LD attenuation for the whole genome. (**c**) Visualization of genetic relationships of each rice variety based on ADMIXTURE analysis.

Each group contained 9, 31, 8, 7, 16, 26, and 26 varieties. The lowest K value was 7 (Figure 3a,c, Table S3). The fourth group contained only *japonica* varieties (R15, R119, R16, R17, R33, R45, and R88). There were two other *japonica* varieties in the second group (R39, R110) and one *japonica* variety in the sixth group (R114). Varieties with resistance were in the second (R08, R14, R19, R24, R25, and R84), first (R89), fourth (R15), fifth (R26, R96), and seventh (R109, R13) groups.

High-quality SNPs with an allowed missing rate of 20% were selected to construct a maximum likelihood phylogenetic tree for the 123 varieties. The results were similar to the mixed analysis (Figure S1, Table S4). In the linkage disequilibrium (LD) attenuation analysis, the average LD of SNPs at a distance of 20 kb was 0.30 (r^2^) and attenuated to half value (~0.30) at a distance of about 233 kb. At the same time, LD attenuation was calculated at the chromosome level. The highest r^2^ (0.33) was found on chromosome 7 and the lowest (0.28) on chromosome 10 (Table S5).

### 3.4. GWAS and Candidate Gene Identification

**Figure 4:**
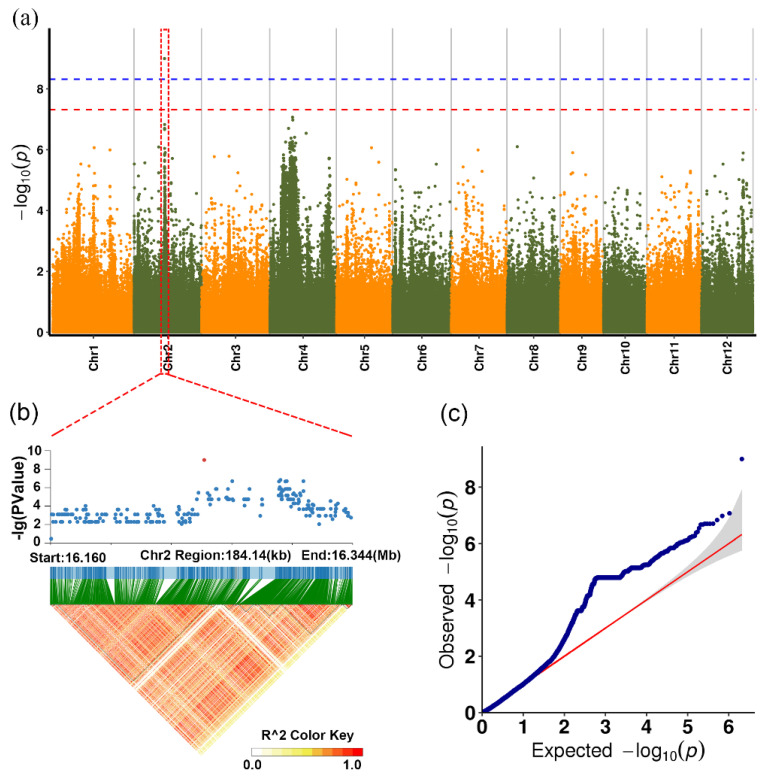
Manhattan plot of genome association analysis and linkage disequilibrium block analysis. (**a**) Manhattan plot of FaST-LMM association mapping. (**b**) LD block module analysis of chromosome 2. (**c**) Graph of expected and observed *p* values.

Approximately 1.89 million SNP/Indel (Indel = insertion/deletion sites) sites were identified. TASSEL, FaST-LMM, and EMMAX software were used for the GWAS. No significant site related to BPH resistance was found. One QTL (quantitative trait locus) related to durable resistance was identified on chromosome 2 by the FaST-LMM model. Genome-wide LD analysis was performed on the candidate peak regions and identified LD modules containing significant SNPs/InDels (regions containing putative candidate genes). LD block analysis was centered on the highest log10(p) values detected in the 200 kb range (Figure 4a). According to annotation of the SNPs/Indels in the upstream and downstream ranges of 200 kb LD block analysis, 13 genes were identified (*LOC_Os02g27430*, *LOC_Os02g27440*, *LOC_Os02g27450*, *LOC_Os02g27470*, *LOC_Os02g27480*, *LOC_Os02g27490*, *LOC_Os02g27500*, *LOC_Os02g27540*, *LOC_Os02g27550*, *LOC_Os02g27560*, *LOC_Os02g27580*, *LOC_Os02g27590*, and *LOC_Os02g27592*). Most of the highly correlated SNPs/Indels were in LD modules of different sizes, indicating significant linkage disequilibrium. Therefore, these candidate genes might function independently or synergistically with other genes containing these SNPs/Indels. Based on the annotation of the 13 genes (Table 2), only six were homologous with *Arabidopsis* genes and the remaining seven were not found in *Arabidopsis*. Of them, seven genes had annotation information. The remaining genes were annotated as hypothetical proteins. *LOC_Os02g27440* coded for DNA topoisomerase, *LOC_Os02g27500* was a leucine-rich protein with an obvious CC-NBS-LRR domain related to disease resistance, and *LOC_Os02g27540* had an NB-ARC domain that was also related to disease resistance. The candidate genes containing many highly correlated SNPs/Indels in the gene region and some highly correlated signals that were not located in known genes were screened (the unknown genes might also be related to durable resistance). Some of the SNPs/Indels were located in the coding region of the unknown genes rather than in the surrounding 200 kb region.

### 3.5. Candidate Gene Expression Analysis

**Figure 5:**
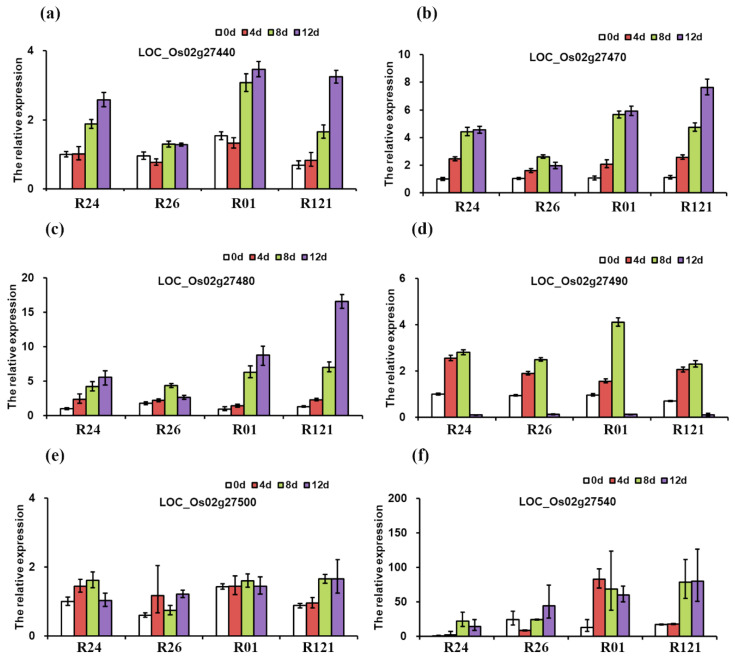
The gene expression changes of six candidate genes after BPH inoculation by qRT-PCR, **namely**, *LOC_Os02g27440* (**a**), *LOC_Os02g27470* (**b**), *LOC_Os02g27480* (**c**), *LOC_Os02g27490* (**d**), *LOC_Os02g27500* (**e**), and *LOC_Os02g27540* (**f**).

In contrast with that at 0 d, the expression level of LOC_Os02g27440 was unchanged at 4 d but up-regulated obviously at 8 d and 12 d after inoculation with BPHs (Figure 5a). The expressions of *LOC_Os02g27470* and *LOC_Os02g27480* were up-regulated obviously after inoculation with BPHs, especially in NDR (R01and R121) (Figure 5b,c). However, the expressions of *LOC_Os02g27440*, *LOC_Os02g27470,* and *LOC_Os02g27480* at 0 d had no apparent difference between LDR (IR24 and R26) and NDR (R01and R121). The expression of *LOC_Os02g27490* was up-regulated at 4 d and 8 d, but it was hardly detected at 12 d (Figure 5d). In addition, the expression of *LOC_Os02g27500* was induced by BPHs to some degree though it had no significant change (Figure 5e). The expression of *LOC_Os02g27540* was up-regulated significantly after inoculation with BPHs in all these rice varieties (Figure 5f). Overall, the expression of these candidate genes was induced by BPHs, and the gene function is worthy of further study.

## 4. Discussion

The BPH is one of the most important pests endangering rice. Hundreds of millions of kilograms of rice are lost every year, seriously threatening rice production and food security [25]. The BPH directly sucks vascular bundle fluid from the rice plant, causing mechanical damage, nutrient loss, and significantly affecting plant growth. Furthermore, it can transmit viruses causing, for example, grassy stunt disease and ragged stunt disease, leading to a severe reduction in rice production or even total crop failure [26]. The traditional method of controlling such damage was to apply chemical pesticides, but this method has obvious disadvantages: primarily the induction of pesticide-resistant BPHs and environmental pollution [27]. Screening and breeding new pest-resistant rice varieties carrying BPH resistance genes are among the most economical and environmentally friendly ways to limit pest damage [28,29].

The Standard Seedbox Screening Technique established by the International Rice Research Institute was a recognized resistance identification method. The later Modified Seedbox Screening Technique made resistance identification more accurate. Seedling screening is still one of the primary methods for identifying resistance to rice planthoppers [30]. In this study, screening was used to test 123 rice varieties at the seedling stage and to identify their durable resistance. Three varieties were immune to BPHs, and nine were highly resistant. Most varieties had no durable resistance period, and less than half were resistant to BPH. The varieties in this study could be divided into seven groups according to evolutionary genetic relationships (Figure 3, Table S2). Further analysis found no obvious correlation between pest resistance and the classification of *indica* and *japonica* subspecies. There was also no apparent genetic pattern among the resistance genes (Figure 2 and Figure S1).

Researchers have analyzed and identified BPH resistance genes since the 1960s. The largest resistance gene clusters are on chromosome 4, with eight genes (*Bph3*, *Bph12*, *Bph15*, *Bph17*(t), *Bph20*(t), *Bph30*, *Bph33*, and *Bph36*) located on the short arm and six (*Bph6*, *bph12*(t), *Bph27*, *Bph27*(t), *Bph34*, and *Bph18*) on the long arm. Eight genes (*Bph1*, *Bph2*, *Bph7*, *Bph9*, *Bph10*, *Bph18*, *Bph21*, and *Bph26*) are located on the long arm of chromosome 12. *Bph13* and *Bph19* are on the short arm of chromosome 3, while *Bph31*, *Bph14* and *Bph11* are on the long arm. Five resistance genes (*Bph25*, *Bph29*, *Bph32*, *Bph4* and *Bph3*) are on the short arm of chromosome 6 [21]. Eight BPH resistance genes (*Bph3*, *Bph6*, *Bph9*, *Bph14*, *Bph26*, *Bph29*, *Bph18*, and *Bph32*) have been successfully cloned [19,31,32,33,34,35]. Four resistance genes (*Bph14*, *Bph26*, *Bph18*, and *Bph9*) contain CC-NBS-LRR domains. *Bph14* was the first resistance gene cloned by map-based cloning and encodes a CC-NBS-LRR domain. It is known that proteins containing CC-NBS-LRR domains can directly or indirectly recognize pathogen-associated molecular patterns or pathogen effectors that induce resistance [19].

One QTL related to the durable resistance of BPH was found on rice chromosome 2 based on the GWAS of 123 rice varieties, and this interval contained 13 genes. Among them, *LOC_Os02g27500* encodes a protein with a CC-NBS-LRR domain, which is the most likely a candidate BPH resistance gene. Out of eight map-based cloned BPH resistance genes, four genes, i.e., *Bph14*, *Bph26*, *Bph18* and *Bph9*, encode CC-NBS-LRR domain-containing proteins. *Bph14* is the first cloned BPH resistance gene that encodes a CC-NBS-LRR domain-containing protein, and its unique LRR domain might function in recognition of the BPH invasion and activating defense response [19]. *Bph26* encodes a CC-NBS-LRR domain-containing protein and mediates resistance by inhibiting BPH from sucking the phloem sap [20]. *BPH18*, located at the same locus of *BPH26*, also encodes a CC-NBS-LRR domain-containing protein that recognizes the BPH invasion at endo-membranes in phloem cells [32]. *Bph9* is another gene encoding the CC-NBS-LRR-containing protein that promotes cell death [35]. The CC domains of BPH9 induce resistance and a hypersensitive response, and the LRR domain of BPH9 confers resistance specificity to BPH [36]. *LOC_Os02g27540* encodes a resistance protein containing a leucine-rich repeat (LRR) and an NB-ARC domain (nucleotide-binding adaptor shared by APAF-1, R proteins and CED-4). The interaction of the internal substructures of the NB-ARC domain is closely related to its activation of downstream anti-disease signaling pathways and functions [37,38]. *LOC_Os02g27430* encodes a GRF zinc finger protein family, which generally plays important roles in plant growth and development, as well as abiotic and biotic stress responses [39,40,41]. In addition, *LOC_Os02g27490* encodes a sodium bile acid symporter family protein, which has been found to be involved in stress resistance [42]. Thus, these candidate genes are probably related to abiotic and biotic stress resistance and may play a role in resistance to the BPH. Among the mapped BPH resistance genes, only *Bph13(t)* is located on chromosome 2, which is located between the SSR markers RM240 and RM250 on the long arm of chromosome 2 and has not yet been cloned [43]. Further analysis showed that the BPH resistance locus identified in this study and *Bph13(t)* are in different regions of chromosome 2. Therefore, the locus found in this study is likely to be a novel resistance region.

In this study, the expressions of six candidate genes were all induced by BPHs to varying degrees. However, the expression of these candidate genes had the same variation trend in both LDR and NDR. The expression levels of these genes were almost the same at 0 d in LDR and NDR, which showed that the expression of these genes had no difference under normal conditions among the different rice varieties. The expressions of *LOC_Os02g27490* and *LOC_Os02g27500* were at the same level at different time periods in LDR and NDR after inoculation with BPHs. In addition, the expressions of *LOC_Os02g27440*, *LOC_Os02g27470*, *LOC_Os02g27480* and *LOC_Os02g27540* were more obviously up-regulated at 8 d and 12 d in NDR compared with those in LDR, especially the expressions of *LOC_Os02g27480* and *LOC_Os02g27540*. The different gene expression patterns indicated different functions of these candidate genes in the resistance to brown planthopper, and the function of genes needs to be further investigated.

It has also been shown that the resistance of plant lines carrying both resistance genes, *Bph14* and *Bph15*, is higher than lines containing only one [44]. Moreover, varieties with a single resistance gene have a shorter service life and are more likely to lose resistance compared to varieties with multiple resistance genes [25]. Therefore, mapping and utilizing new resistance genes, pyramiding multiple resistance genes into one variety, and breeding rice varieties with durable resistance are key to promoting BPH resistance. In this study, a new BPH resistance QTL was identified through a GWAS. In a future study, the function of candidate genes will be analyzed to determine their roles in resistance, in the hope of providing new genetic resources for breeding BPH-resistant rice varieties.

## 5. Conclusions

In this study, we quantified the BPH tolerance of 123 rice varieties and reported several highly tolerant rice varieties. These highly tolerant varieties provide a valuable gene pool for tolerance breeding of BPH in rice. Here, a new BPH resistance QTL was identified through a GWAS. Our finding will benefit the understanding of the molecular mechanism of BPH in rice and the provided novel QTLs/gene targets for breeding improved BPH tolerance of rice varieties through gene editing.

## Figures and Tables

**Table 1:** Distribution of single nucleotide polymorphisms (SNPs) across 12 rice chromosomes.

Chromosome	Number of SNPs	Length of Chromosome (bp)	Density of SNP (bp/SNP)
1	231,924	44,136,341	190
2	204,384	37,374,738	182
3	209,755	37,870,371	180
4	167,776	36,922,800	220
5	143,995	31,156,770	216
6	172,799	32,498,737	188
7	83,242	30,885,525	371
8	146,953	29,580,742	201
9	121,029	23,933,228	197
10	130,727	24,135,577	184
11	142,287	30,181,950	212
12	142,974	29,899,725	209
Total	1,897,845	388,576,504	204

**Table 2:** Annotation of candidate rice genes related to durable brown planthopper resistance.

Gene ID	Arabidopsis ID	Description
*LOC_Os02g27430*	NA	GRF zinc finger family protein
*LOC_Os02g27440*	AT4G31210.1	DNA topoisomerase, type IA, core
*LOC_Os02g27450*	NA	expressed protein
*LOC_Os02g27470*	AT2G31660.1	ARM repeat superfamily protein
*LOC_Os02g27480*	AT1G14780.1	MAC/Perforin domain-containing protein
*LOC_Os02g27490*	AT3G25410.1	Sodium Bile acid symporter family
*LOC_Os02g27500*	AT5G43470.2	Disease resistance protein (CC-NBS-LRR class) family
*LOC_Os02g27540*	AT3G14460.1	LRR and NB-ARC domains-containing disease resistance protein
*LOC_Os02g27550*	NA	hypothetical protein
*LOC_Os02g27560*	NA	expressed protein
*LOC_Os02g27580*	NA	hypothetical protein
*LOC_Os02g27590*	NA	hypothetical protein
*LOC_Os02g27592*	NA	expressed protein

## Data Availability

All sequence data of the samples are available at the NCBI SRA (Sequence Read Archive) database website under accession number PRJNA743713.

## Supplementary Materials

The following are available online at https://www.mdpi.com/article/10.3390/insects12090836/s1, Figure S1: Phylogenetic tree of 123 rice varieties based on single nucleotide polymorphisms (SNPs). The three BPH-immune rice varieties are indicated by blue dots, and the japonica rice varieties are indicated by green dots, Table S1: Primers used in this study, Table S2: Sample sequencing depth statistics, Table S3: The K value of all samples using an admixture, Table S4: Corresponding table of the evolutionary tree, Table S5: Linkage disequilibrium decay value.
