# Supplementary figures and images for: Genome-Wide Association Study Reveals a New Quantitative Trait Locus in Rice Related to Resistance to Brown Planthopper Nilaparvata lugens (Stål)
Source: Insects. 2021 Sep 17;12(9):836. doi: 10.3390/insects12090836 (PMC8469741; doi:10.3390/insects12090836)

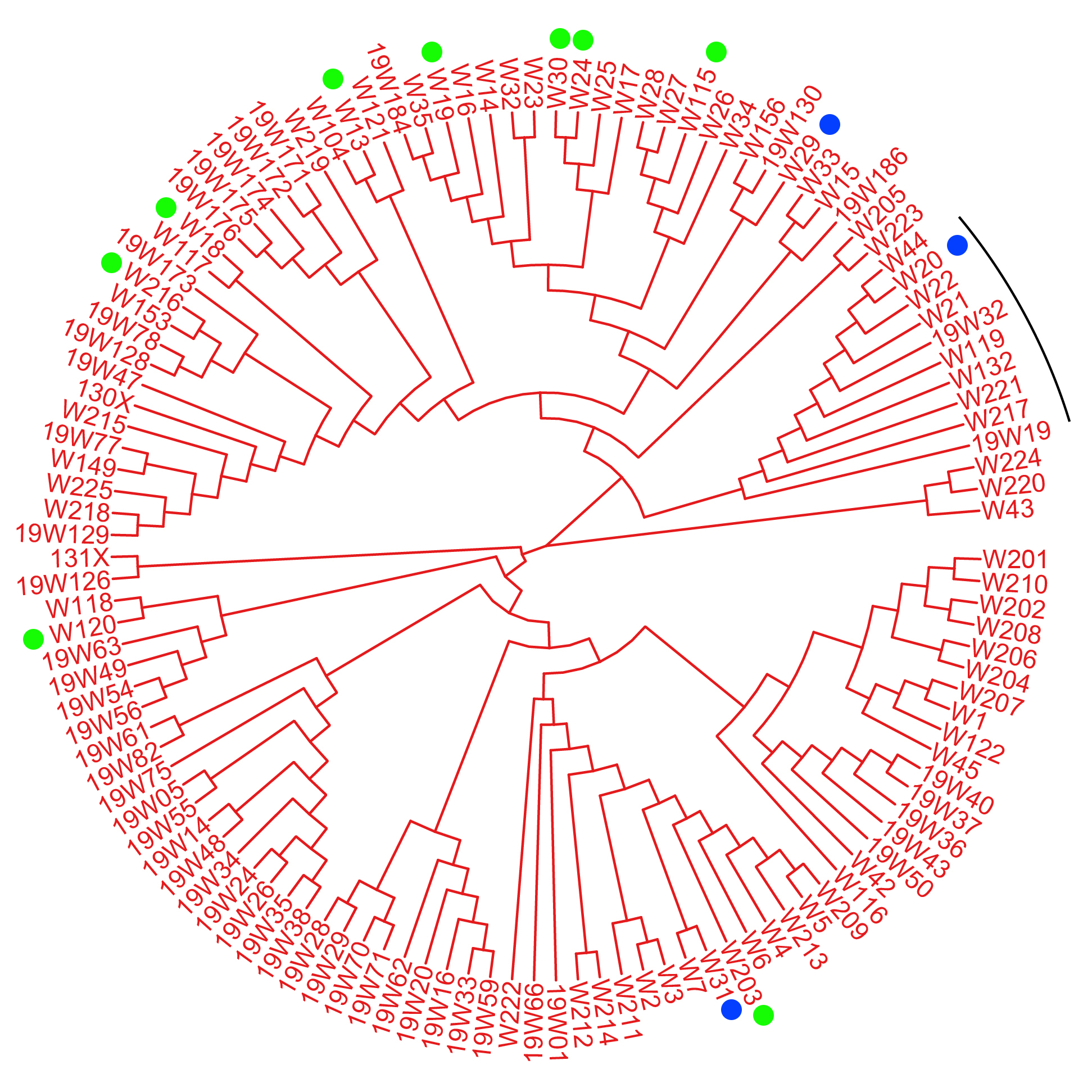

Supplement: Supplementary file 1 [file insects-12-00836-s001.zip › Figure S1.jpg]
